# Caregivers’ acceptance of alternatives to long-term psychiatric hospitalization; lessons and debates from the South Korean situation

**DOI:** 10.1186/1752-4458-8-4

**Published:** 2014-01-17

**Authors:** Myung-Soo Lee, Jong-Ik Park

**Affiliations:** 1 Seoul Mental Health Center, Seoul, Republic of Korea; 2 Yongin Mental Hospital, Yongin, Republic of Korea; 3 Department of Psychiatry, Kangwon National University School of Medicine, 156 Baengnyeong-ro, Chuncheon 200-947, Korea

## Abstract

**Background:**

A political movement towards building alternatives to long-term hospitalization of psychiatric patients in Korea has gained momentum. We aimed to provide sturdy foundation needed to formulate the most rational policy by review of caregiver’s opinion to the political alternatives under discussion for facilitating discharge of long-term stayed psychiatric patients in Korea.

**Discussion:**

Caregivers in Korea, whose family members had been hospitalized longer than 6 months and all of whom applied to the Mental Health Review Board (MHRB) for an examination required for extended stay, have shown reluctance to take their patients back home. Especially, a half of them answered that if MHRB would order compulsory discharge, they would take their patients to another hospital instead of living together. Despite of those pessimistic attitudes, one of the promising solutions might be residential care as an alternative to the long-term hospital care, which is most preferred by caregivers.

**Conclusion:**

After all, the issue of who should take an accountability of the psychiatric patients is essential in establishing mental health policy. Korean government should analyze and reform mental health delivery systems such as residential service system, community-based case management programs and hospital treatment systems including payment program which can facilitate reasonable decision by professionals as well as caregivers for the appropriate admission rather than longer term hospitalizations.

## Background

“Mental Health in Korea: OECD Review and Recommendation” [1] criticizes the nation's issue surrounding its treatment of psychiatric patients: excessively long hospitalization and involuntary admittance by family and other caregivers. The report proceeds and identifies the unavailability of alternatives–community services for instances–as the core of the problem. It urges for an active movement away from the country’s institutionalization-based service provision system towards a community-based one. The Mental Health Act [2] enables the legal and political mechanisms promoting i) discharge of involuntarily-hospitalized psychiatric patients, and ii) protection of their rights. Mental Health Bill 2013 proposes reduction of the time frame before assessment for extended hospitalization from its original six months to two months.

This domestically–and internationally-recognized political issue in Korea surrounding long-term inpatient care of psychiatric patients is grave. In 2011, the median duration of hospital stay was 160 days, 271 days in private hospitals, and over seven years in asylums [3]. The government proposed National Mental Health Five-Year Plan as a political solution to this matter [4]. However, its prospective effect on the caregivers of psychiatric patients under long-term hospitalization remains unclear. Apart from the statistical correlations between long-term stays and series of independent variables derived from previous studies, the caregivers’ reactions to the proposed government policies have not been thoroughly investigated.

This survey examined compliance of psychiatric patients’ caregivers to the alternatives to long-term hospitalization currently under discussion. In doing so, we hope to aid in establishing the most rational policy that would mitigate the long-term hospitalization matter.

## Discussion

### The caregivers’ responses concerning alternatives to long-term hospitalization

We targeted psychiatric patients’ caregivers who have applied to the Mental Health Review Board (MHRB) for an examination required for continued hospitalization. We selected caregivers from one public and one private mental hospital in Gyeonggi province who applied to the MHRB from January to December of 2008 and from all of psychiatric hospitals in the city of Seoul from October to December of 2008.

The telephone survey was done by social workers from the two selected hospitals in Gyeonggi province and by trained mental health professionals in Seoul city. Cumulatively, of those 475 candidates from Seoul (196) and Gyeonggi province (279), 208 (81 from Seoul and 127 from Gyeonggi) were contacted, and of them, 136 (72 from Seoul and 127 from Gyeonggi) completed the questionnaire and the ultimate rate of participation was 28.6%. This survey was approved by Institutional Review Board of Seoul Municipal Eunpyeong Hospital.

The questionnaires included brief psychiatric history, socio-relational characteristics and caregivers’ willingness to acceptance of discharge regarding on the alternatives such as free residential facilities, day care programs, financial assistance, transfer to national mental hospitals, outpatient treatment order and discharge orders from Mental Health Review Board.

Among the caregivers who completed the survey, siblings were most frequent (48.1%). Parents were 32.6%, children were 7.4%, and spouses were 7.4. The type of medical support was in descending order of frequency, Medical Aid Category I (98, 72.1%), health insurance (30, 22.1%), and Medical Aid Category II (8, 5.9%). The patients’ number of previous admission was one time (20 patients), 2-5 times (52 patients), 6-10 (26 patients) and over 10 times (38 patients). 77.2% of the patients had been hospitalized for over one year.

Caregivers’ opinions on the alternatives expected to facilitate patients’ discharge from psychiatric hospitals are summarized in Table 1.

**Table 1:** Questions and caregivers’ responses concerning alternatives to long-stay hospitalization

**Do you willingly consider your patient’s discharge if government provide or choose following alternatives to hospitalization?**		
Alternative to hospitalization	Yes (%)	No (%)
Free residential facility	49.2	50.8
Financial support if caregiver live with their patient	32.8	67.2
Discharge order from Mental Health Review Board (MHRB)	36.4	63.6
*if the patient was discharged by MHRB, what would you do?*		
*maintain outpatient treatment*	23.5	
*transfer to other mental hospital*	50.0	
*send them to residential facility*	17.7	
*etc*	8.8	
Transfer to public hospital in severe cases	61.1	38.9
Free day care program	32.3	67.7

### Implications to the Korean mental health system

This study’s limitations were as follows. First, the sample population was not fully representative of caregivers of psychiatric patients who were hospitalized long-term in Korea. However, due to the sensitive nature of the research topic, obtaining a fully-representative sample might have been close to impossible. Second, the fragile essence of this survey yielded a response rate of below 30%. This could imply that, although this study’s results show a low compliancy to the proposed alternatives, the actual compliance rate might be even lower than what the findings suggested. Third, their lack of cooperation made it difficult to sufficiently survey the caregivers through a telephone poll. As such, the clinical factors of the corresponding psychiatric patients could not be objectively evaluated, and thus we relied upon the subjective responses given by their caregivers. Because of the data set was not fully complete, the factors affecting caregivers’ compliance to the policies could not be statistically analyzed.

However, despite these limitations, these results offered great insights. This study focused on the caregivers’ responses to policies that were proposed to improve current long-term hospitalization situation. As the results indicated, in general, the caregivers requesting continuation of hospitalization to Mental Health Review Board did not respond positively to the government policies. However, these results did not suggest all Korean caregivers felt this way, and we believe even those who did not comply with the policies would have had put in reasonable efforts during past progressions of the illnesses. Even so, the results indicating only 15% and 53% of the patients had, respectively, just one and under five past experience of hospitalization are worth re-examining. Park et al. [5] states in the first three years of psychosis episodes, 18% of patients have been hospitalized long-term for over six months. This high long-term in-patient experience rate in Korean psychiatric patients who are in their initial stages of treatment is an important issue to re-examine.

Eighty percent of the participants cared for psychiatric patients who received Medical Aid. The high rate of long-term hospitalization in Medical Aid recipients could because the disease characteristics became chronic or because the inpatient treatment did not impose great financial burden on the caregivers. However, financial factors were not panacea. The results revealed that a mere 33% responded positively when financial assisted was offered as an alternative to long-term psychiatric inpatient care.

As of date, the governmental solutions are not accommodated by economics-based policies on medical insurance fees. As such, the Mental Health Review Board and its operations may be the only regulatory system granted by the Mental Health Act. However, for the applicants for continuation of hospitalization, the rate of decree to discharge ceased at 4% in 2011 [6]. According to a study by Lee et al. [7], of the patients who were released from the hospital upon the discharge order from the Review Board, 28% of them were re-admitted to hospitals within a day. Similarly, in this study, when asked about discharge ordered by the Review Board, 64% responded negatively and 50% replied they would admit their patients to a different hospital if MHRB ordered discharge.

An important issue in such situations may be who should take an accountability of the psychiatric patients. The responsibilities once within the family system in agrarian society were weakened with the beginning of the nuclear family era. Previous studies conducted in Korea reveal the length of hospital stay becomes significantly longer with a decrease in the level of family support [8-10]. Compared to other developed countries, Korean societal responsibility has not reached its maturity. Because the government cannot obtain full accountability of psychiatric patients, families still have great responsibilities and rights over them as their custodians. For instance, in western countries, families only have the right to request for their patients’ admittance to hospitals. On the contrary, in Korea, families actually have the authority to determine the admission as well as the discharge of those under their care. At this point, almost the only role that could be taken on by the country is providing financial support for Medical Aid patients’ hospital stay. This governmental assistance attracts the patients.

If a rational mental health delivery system was defined as one that ultimately gathers patients at a suitable service location, for psychiatric patients who receive long-term inpatient care due to their lack of social support system, housing services would be an essential element needed for their return to the community. Similarly, in this study, residential facilities yielded most compliance from the caregivers. By the standards of the year 2012, national providers of residence for mental illness are established in 160 places, and an estimated total capacity for 2,100 patients. However, these service providers are installed in only 35% of the 232 national districts, and almost 40% of these total housing facilities are concentrated in City of Seoul [4]. So in reality, accessibility to these services is extremely insufficient. Adequate supply strategy, where the establishment and promotion of the housing services are in accordance with their demands, may be needed.

## Conclusion

Korean government should reform mental health delivery systems such as residential service system, community-based case management programs and hospital treatment systems including payment program which can facilitate reasonable decision by professionals as well as caregivers for the appropriate admission rather than longer term hospitalizations.

## Competing interests

The authors declare that we have no competing interests.

## Authors’ contributions

M-S L educated and supervised mental health professionals who had carried out telephone survey and also summarized survey results, and wrote the manuscript. J-I P designed this survey and made concept of this article and also wrote this manuscript while discussing with M-S L. Both authors read and approved the final manuscript.
